# Efficacy of Local Oxygen Therapy Combined With Human Albumin for Stage 2 Pressure Ulcers: A Case Report

**DOI:** 10.1002/puh2.70173

**Published:** 2025-11-27

**Authors:** Fengrong Tang

**Affiliations:** ^1^ Department of Nursing, Zhongshan Hospital (Xiamen) Fudan University Xiamen Fujian China; ^2^ Department of Nursing Xiamen China

**Keywords:** case reports, human albumin, local oxygen therapy, nursing, pressure ulcers

## Abstract

This study summarizes the nursing management of a patient with cerebral infarction and sacrococcygeal Stage 2 pressure ulcer, focusing on the effectiveness of local oxygen therapy combined with human albumin. Patients are evaluated for the risk of pressure ulcers and nutritional deficiencies upon admission. We applied topical oxygen therapy combined with human albumin for dressing changes, during which the healing of pressure ulcers is assessed promptly. The pressure ulcer completely healed within 1 week, The new method of pressure ulcer care, which combines local oxygen therapy with human blood albumin, has been shown to shorten wound healing time, enhance nursing effectiveness, and provide a more efficient and feasible nursing technology for the clinical promotion of pressure ulcer management.

## Graphical abstract

The Numerical Rating Scale(NRS) is a self‐report tool for assessing pain intensity.It uses an 11‐point scale from 0 to 10, where 0 represents “no pain ” and 10 represents the most severe pain. The scale includes verbal descriptors for ranges of scores:mild, moderate, and severe pain, aiding in standardized clinical communication and assessment.

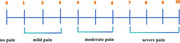

## 1 Introduction

A pressure ulcer is a condition resulting from prolonged tissue compression, leading to ischemia, hypoxia, and malnutrition, which ultimately causes tissue ulceration and necrosis. Elderly bedridden patients with cerebral infarction are at a heightened risk of developing pressure ulcers, which can be challenging to heal once they occur. These ulcers are a prevalent condition that significantly impacts the quality of life for elderly patients and poses a serious threat to the lives of those with cerebral infarction [1, 2]. Research indicates that the annual cost of treating pressure ulcers is estimated to be between $11 billion and $17.2 billion in the United States, £1.4 billion to £2.1 billion in the United Kingdom, and approximately 296.05 million Australian dollars in Australia [3, 4, 5]. Numerous treatment methods for pressure ulcers are available both domestically and internationally, including physical, biological, and integrated traditional Chinese and Western medicine approaches; however, their efficacy varies. Local oxygen therapy creates a hyper‐oxygenated environment that enhances wound healing by promoting tissue oxygenation and reducing bacterial proliferation [6]. Human albumin is a blood product that contains a variety of proteins essential for the human body. It can be applied topically to ulcer surfaces to promote the growth of granulation tissue, enhance the osmotic pressure of local tissues, reduce local edema, minimize exudation from the ulcer surface, and protect the ulcer itself [7, 8]. On July 10, 2024, our department admitted a patient with cerebral infarction and a Stage 2 sacrococcygeal pressure ulcer. We effectively combined the aforementioned methods, utilizing a novel approach of local oxygen therapy along with human blood albumin for the treatment of pressure ulcer wounds. This strategy significantly shortened the wound healing time and improved patient care outcomes, providing a more effective and feasible nursing technology for the clinical promotion of pressure ulcer healing.

## 2 Case Presentation

The patient, an 88‐year‐old male with a height of 175 cm, a weight of 83 kg, and a BMI of 27.1 kg/m^2^, has a history of hypertension, atrial fibrillation, and cerebral artery stenosis. He was admitted to the hospital on July 10, 2024, due to “speech inability with right limb weakness for more than 3 h.” Upon admission, the patient was conscious. Physical examination revealed non‐fluent speech and partial comprehension difficulties. Muscle strength in the right limb was rated at V+, with slightly reduced sensation and positive pathological signs. The NIHSS score was 2. Vital signs upon admission were as follows: *T*: 36.5°C, *P*: 69 bpm, *R*: 20 breaths/min, BP: 188/86 mmHg. Test results indicated: glucose: 6.1 mmol/L; carbon dioxide: 22 mmol/L; lipase: 99.6 U/L; total protein: 58 g/L; albumin: 31 g/L; globulin: 27 g/L; platelet count: 99 × 10^9^/L; white blood cell count: 4.31 × 10^9^/L; percentage of neutrophils: 52.5%. The patient has no history of smoking or alcohol consumption. A Stage 2 pressure ulcer measuring 3 × 2.5 cm^2^ was noted in the sacrococcygeal region.

**FIGURE 1:**
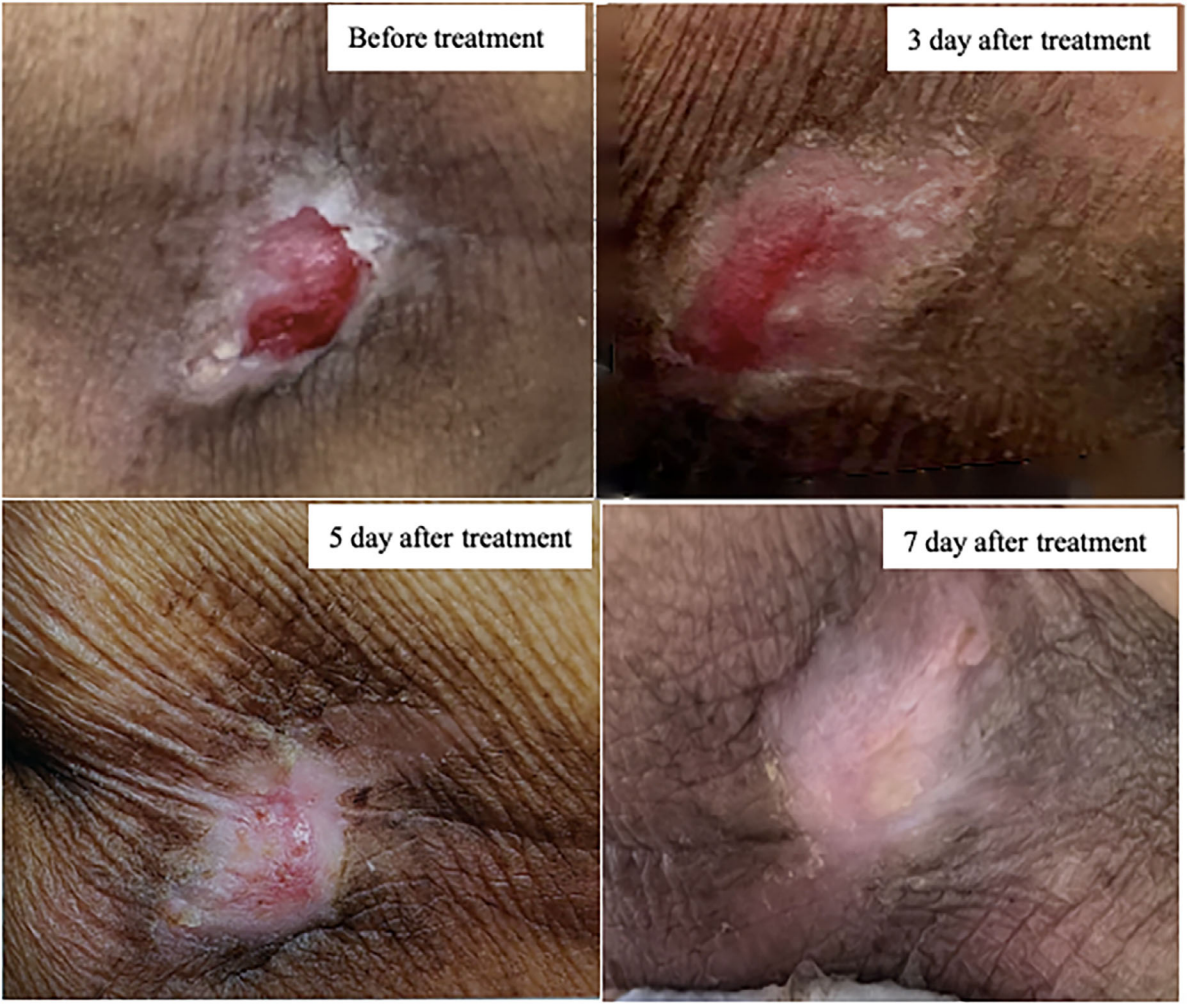
The healing time for the pressure ulcer completely resolves after 7 days of dressing changes, as illustrated in the figure.

After admission, the patient received treatment, including anti‐infection measures, anti‐thrombosis therapy, lipid regulation, stabilization of vital signs, improvement of circulation, gastric protection, and nutritional support. Blood pressure was monitored and controlled. Local oxygen therapy was applied to the pressure ulcer in conjunction with human albumin dressing changes. The ’patient's pressure ulcer was completely healed after 7 days of dressing changes (Figure 1), and no new pressure ulcers developed. There were no signs of infection at the wound site. The wound dressing change and care methods were as follows.

### 2.1 First, the Wound Was Dressed According to Surgical Aseptic Principles

The wound was flushed with normal saline, and necrotic tissue around the pressure ulcer, along with devitalized skin, was excised using sterile scissors. The surrounding skin was disinfected with 0.5% iodophor, and the wound was wiped with a 0.9% sodium chloride cotton ball to remove secretions. Subsequently, the wound was irrigated with 20 mL of 0.9% sodium chloride using a vortex flushing technique.

### 2.2 Second, Local Oxygen Therapy Was Applied to the Wound

This commonly used technique in clinical practice promotes the formation of a protective crust on the wound surface, enhances oxygen content in the tissue, inhibits bacterial reproduction, and accelerates wound healing [9]. After each dressing change and protein application, an oxidized nebulized catheter was connected to the wound to deliver oxygen. The oxygen flow rate was adjusted to 5 to 8 L/min, administered 1 or 2 times a day for 10–15 min each session.

### 2.3 Lastly, Human Blood Albumin Was Applied to the Wound Following Local Oxygen Therapy

A cotton swab was used to evenly distribute the human blood albumin solution over the wound (if the solution was insufficient, it could be diluted with saline). Oxygen was then blown onto the wound for approximately 5 min until the albumin formed a transparent film, after which Mepilex was applied.

At admission, the assessment was based on weight, age, body mass index, upper arm muscle circumference, triceps skinfold thickness, protein levels, immune status, nitrogen balance, and other relevant factors. The patient″s serum albumin level was 31 g/L (normal range: 35 g/L or higher), and the total score on the NRS 2002 [10] was 5, indicating nutritional risk (Table 1).

**TABLE 1:** Nutritional risk screening, NRS2002.

A. Nutritional impairment (any)		B. Disease severity score (either)	
Normal (0 points)	Normal nutritional status		
Mild (1 point)	Weight loss of >5% in the past 3 months and a reduction in food intake by >25% in the past week	Mild (1 point)	Pelvic fracture or chronic disease with the following conditions: liver cirrhosis, COPD, long‐term hemodialysis, diabetes, cancer
Moderate (2 points)	Weight loss of >5% in the past 2 months and a reduction in food intake by >50% in the past week	Moderate (2 points)	Major abdominal surgery, stroke, severe pneumonia, hematologic malignancy
Severe (3 points)	Weight and mass loss of >5% in the past month, a decrease in eating by >75% in the past week, BMI < 18.5 kg/m^2^, and poor general condition	Severe (3 points)	Head injury, bone marrow transplant, ICU patients (APACHE II > 10 points)

*Note:* Age: A score of 1 point is added if the patient is ≥70 years old. Total score = nutritional score + disease score + age score. If the total score is ≥3: The patient is at nutritional risk, and nutritional support should be initiated. If the total score is <3: Weekly reassessment of nutrition is required; prophylactic nutritional support should be considered if the patient is undergoing major surgery.

The Pressure Ulcer Scale for Healing, PUSH, [11] is designed to monitor and document the progress of wound healing (Table 2). The patients″ scores were recorded as follows: 10 points before treatment, 9 points 1 day after treatment, 6 points 3 days after treatment, 3 points 5 days after treatment, and 1 point 7 days after treatment.

**TABLE 2:** Pressure ulcer scale for healing, PUSH.

Scoring items	Scoring content	Scoring criteria
Wound area (cm^2^)	0	0
	<0.3	1
	0.3–0.6	2
	0.7–1.0	3
	1.1–2.0	4
	2.1–3.0	5
	3.1–4.0	6
	4.1–8.0	7
	8.1–12.0	8
	12.1–24	9
	>24	10
24‐h exudate (mL)	Dry and non‐exuding	0
	<5 mL (small amount)	1
	5–10 mL (medium amount)	2
	>10 mL (large amount)	3
Type of wound tissue	Close	0
	Superficial with epithelial tissue growth	1
	Clean with granulation tissue growth	2
	Contains necrotic tissue but not significant	3
	Contains significant necrotic tissue	4

The Numerical Rating Scale, NRS, [12] is a single‐dimensional pain scale used to assess both acute and chronic pain, including postoperative pain, cancer pain, and arthritis pain (Table 3). The scores recorded were: 6 points before treatment, 6 points 1 day after treatment, 3 points 3 days after treatment, 1 point 5 days after treatment, and 0 points 7 days after treatment.

**TABLE 3:** Numerical rating scale (NRS).

Classification	Score	Symptoms
No pain	0 points	Indicates that the patient is not experiencing any pain
Mild pain (does not affect sleep)	1–3 points	1 point: No pain when lying quietly, pain when turning over or coughing; 2 points: Pain when coughing, deep breathing is not painful; 3 points: No pain when lying quietly, pain occurs with coughing and deep breathing
Moderate pain (sleep is shallow)	4–6 points	4 points: Pain is present when lying quietly; 5 points: Persistent pain when lying quietly; 6 points: severe pain when lying still
Severe pain (sleep is severely disturbed)	7–10 points	7 points: Severe pain, restlessness, fatigue, and inability to sleep; 8 points: Persistent pain with profuse sweating; 9 points: severe pain that is unbearable; 10 points: the most intense pain, feeling that life is worse than death

*Note:* The patient's pain level is assessed on the basis of the number selected by the patient. Different numeric ranges correspond to varying levels of pain, as detailed in this table. In the NRS scoring system: no pain (0 points), mild pain (1–3 points), moderate pain (4–6 points), and severe pain (7–10 points).

## 3 Nursing

### 3.1 Observation of the Condition

Careful observation of the patient″s condition is essential, with timely and accurate documentation of any changes. The wound is assessed at each dressing change, and nurses on each shift must attentively monitor both the patient″s overall condition and the state of the wound, ensuring effective bedside handovers.

### 3.2 Basic Care

Maintain continuous inflation of the air cushion bed and assist the patient in repositioning every 2 h. It is advisable for the patient to adopt a left or right lateral decubitus position, with a tilt of 30°–45° when lying on their side. A turning pillow should be placed against the patient″s back to prevent pressure sores on the sacrococcygeal area. Care should be taken to position the patient″s limbs correctly to mitigate poor habits and prevent complications, such as foot valgus [13], drooping, and shoulder subluxation. This approach will enhance the treatment outcome and quality of life for patients with cerebral infarction. Ensure that bed linens are clean, flat, wrinkle‐free, and free of debris to minimize friction and utilize lift sheets to assist the patient in moving around the bed.

### 3.3 Nutritional Support

Patients with cerebral infarction often experience prolonged recovery due to decreased mobility, neurological function, and sensory perception, leading to reduced tissue metabolism and extended healing times [14]. Therefore, enhancing nutritional intake and immune support, actively treating underlying conditions, and managing infections are critical for effective pressure ulcer treatment [15]. The patient was provided with a nasogastric tube 3 days post‐admission and received daily enteral nutrition, consisting of a mixed suspension (full force) and enteral nutrition powder. By implementing early screening and assessment for nutritional risks, healthcare providers can monitor the patient″s nutritional status and address malnutrition promptly. A multidisciplinary team conducts a comprehensive evaluation of the patient and formulates a nutritional support plan aimed at improving the patient″s nutritional status.

### 3.4 Psychological Care

Given the extensive nature of pressure ulcers and the associated pain, analgesics should be administered as necessary to alleviate discomfort. Providing psychological support is crucial; it is important to encourage patient engagement, communicate with family members, and keep them informed after each dressing change to alleviate anxiety regarding the wound. Educating patients and their families about the related conditions, pressure ulcer prevention, and care methods is essential. Frequent encouragement helps build the patient″s confidence in overcoming their condition. During hospitalization, the patient exhibited emotional stability and actively cooperated with medical staff, leading to prompt control of the disease.

## 4 Discussion

Elderly patients with long‐term bed rest have the highest incidence of pressure injuries. A multicenter study found that the incidence of pressure ulcers among hospitalized elderly adults ranged from 10% to 25% [16]. The healing of pressure injuries involves various tissue regeneration processes, including granulation tissue hyperplasia and scar formation, which are complex and orderly biological processes [17]. Traditional treatment methods include protecting local wounds, changing dressings, combating infections, and enhancing nutritional support. According to biological principles of healing, identifying new methods to promote the healing of pressure ulcers holds significant clinical and social value. This case study involves a single patient, resulting in a relatively small sample size; thus, the study results may vary. Additionally, there are no relevant studies addressing the contraindications and side effects of topical human albumin, but we will explore and monitor these aspects in subsequent studies. Ye et al. [18] analyzed the clinical effects of human albumin in treating pressure ulcers, revealing that patients in the observation group exhibited higher efficacy compared to the control group after using human albumin. The healing time for the observation group was (14.07 ± 2.09) days, significantly shorter than the control group″s healing time of (20.03 ± 2.11) days, with a statistically significant difference (*p* < 0.05). Compared to the control group, which used conventional pressure ulcer powder gauze to cover the wound surface, human albumin demonstrated a significant effect in treating pressure ulcers and was straightforward in clinical application. It can accelerate the healing of the sore surface, alleviate patient pain, and reduce the financial burden on patients. Cheng et al. [19] found that the nursing effect of moxibustion combined with oxygen therapy and hydrocolloid dressing on Stage II and III pressure ulcers resulted in a shorter wound healing time in the observation group (6.43 ± 1.52 days) compared to the control group (10.25 ± 2.23 days), with a statistically significant difference (*p* < 0.05). This study accelerates wound healing compared to conventional wound care methods combined with external hydrocolloid dressing applications.

We applied topical oxygen therapy combined with human albumin during dressing changes. Local oxygen therapy can improve wound hypoxia, promote faster granulation tissue growth, increase capillary oxygen content, and facilitate wound drying and scab formation, thereby accelerating epithelialization and achieving healing [20]. Human albumin is a biological agent [21] with high nutritional and immune effects, capable of maintaining plasma colloid osmotic pressure and regulating the dynamic balance between tissues and blood vessels. As a highly permeable colloid, it can form a protective film when applied to the wound surface, keeping it clean and preventing direct bacterial contamination. Due to the high cost of albumin, a small amount of the stock solution can be retained in the bottle after intravenous administration, allowing for its collection and subsequent use in treating pressure ulcers. This approach not only minimizes waste but also provides an effective treatment option for pressure ulcers that deserves clinical promotion and application.

## Author Contributions

Fengrong Tang is responsible for statistical analysis, paper writing, and clinical implementation.

## Ethics Statement

This study has been approved by the Ethics Committee of Xiamen Hospital, Zhongshan Hospital (Xiamen), Fudan University (B2024‐019), and the patient has signed a written informed consent form.

## Consent

Written informed consent was obtained from the patient for the publication of the patient″s data/images included in this case report.

## Conflicts of Interest

The authors declare no conflicts of interest.

## Data Availability

The authors confirm that the data of this study are publicly available without restriction and available within the article and its supplementary materials.
